# Formulation and *in vivo* assessment of terconazole-loaded polymeric mixed micelles enriched with Cremophor EL as dual functioning mediator for augmenting physical stability and skin delivery

**DOI:** 10.1080/10717544.2018.1436098

**Published:** 2018-02-07

**Authors:** Wessam H. Abd-Elsalam, Sally A. El-Zahaby, Abdulaziz M. Al-Mahallawi

**Affiliations:** a Department of Pharmaceutics and Industrial Pharmacy, Faculty of Pharmacy, Cairo University, Cairo, Egypt;; b Department of Pharmaceutics and Pharmaceutical Technology, Faculty of Pharmacy and Drug Manufacturing, Pharos University in Alexandria, Alexandria, Egypt

**Keywords:** Pluronic P123, Pluronic F127, histopathology, skin deposition, Cremophor EL

## Abstract

The aim of the current study was to formulate terconazole (TCZ) loaded polymeric mixed micelles (PMMs) incorporating Cremophor EL as a stabilizer and a penetration enhancer. A 2^3^ full factorial design was performed using Design-Expert® software for the optimization of the PMMs which were formulated using Pluronic P123 and Pluronic F127 together with Cremophor EL. To confirm the role of Cremophor EL, PMMs formulation lacking Cremophor EL was prepared for the purpose of comparison. Results showed that the optimal PMMs formulation (F7, where the ratio of total Pluronics to drug was 40:1, the weight ratio of Pluronic P123 to Pluronic F127 was 4:1, and the percentage of Cremophor EL in aqueous phase was 5%) had a high micellar incorporation efficiency (92.98 ± 0.40%) and a very small micellar size (33.23 ± 8.00 nm). Transmission electron microscopy revealed that PMMs possess spherical shape and good dispersibility. The optimal PMMs exhibited superior physical stability when compared with the PMMs formulation of the same composition but lacking Cremophor EL. *Ex vivo* studies demonstrated that the optimal PMMs formula markedly improved the dermal TCZ delivery compared to PMMs lacking Cremophor EL and TCZ suspension. In addition, it was found that the optimal PMMs exhibited a greater extent of TCZ deposition in the rat dorsal skin relative to TCZ suspension. Moreover, histopathological studies revealed the safety of the optimal PMMs upon topical application to rats. Consequently, PMMs enriched with Cremophor EL, as a stable nano-system, could be promising for the skin delivery of TCZ.

## 1. Introduction

Skin infections, triggered by different fungal species such as Candida and Trichophyton species, have recently spread worldwide. Fungal infections are more frequent in immunocompromised patients due to cancer chemotherapy, organ transplantation, or human immunodeficiency virus infections (Kim et al., 2008). These infections are the causes of around 1,500,000 deaths every year. Fungal diseases are mainly treated by the oral administration and/or topical application of antifungal agents. Systemic treatments with oral antifungal agents are known to be more effective, however; they cause many toxic side effects and can increase the risk of drug interactions (Young, 1997). Therefore, the development of an advanced topical treatment for fungal infections will be superior in its outcomes owing to targeting the site of infection and minimizing the risk of systemic adverse effects (Güng, 2013). Many approaches were formerly used to enhance the anti-fungal skin permeation namely; stratum corneum modification by the inclusion of an absorption enhancer like dimethyl sulfoxide (Morrow et al., 2007), stratum corneum bypassing by aid of micro-needle array (Nalluri et al., 2017; Pamornpathomkul et al., 2017), utilization of vesicular carriers like liposomes (Touitou et al., 2000; Doppalapudi et al., 2017), nanoparticles approach (Ahad et al., 2017; Sun et al., 2017), pro-drug approach (Bonina et al., 2001; Majumdar & Sloan, 2007), and the energy driven methods like iontophoresis (Huber et al., 2015).

Besides the development of efficient dosage forms of the highly potential available anti-fungal agents; the lately sudden increase in the use of antimycotic drugs resulted in the emergence of resistant strains. Hence an urgent medical need for novel antifungals has been arised. Terconazole (TCZ) is a new broad-spectrum antifungal agent that belongs to the class of triazoles and is mainly used for the treatment of vulvovaginal candidiasis. The main mechanism of action of TCZ simulates that of imidazoles through inhibition of the fungal cytochrome P-450 but it is documented to be more active than imidazoles (Cauwenbergh & Vanden, 1989). The poor permeability characters of TCZ limit its clinical use. To overwhelmed this problem, TCZ were incorporated in bilosomes (Abdelbary et al., 2016) and proniosomal gel (Abdou & Ahmed, 2016).

Mixed nanomicellar carriers are nanoscopic structures (<100 nm), formed by amphiphilic block copolymers. These copolymers are composed of hydrophilic and hydrophobic chains that self-assemble in water, above a certain concentration named the critical micelle concentration (CMC; Chiappetta & Sosnik, 2007). The systems are characterized by their superior solubilizing capacity when compared with the regular micelles owing to their larger cores (Kwon, 2003). In addition, surfactants can disrupt the packing of lipids and proteins within the stratum corneum which is the main barrier for drug penetration, hence, facilitating drugs permeation through skin (James-Smith et al., 2011). Therefore, the nano-sized carriers supplemented with surfactants can present a very promising formulation approach for the topical application of a poorly soluble drug like TCZ.

The current study is the first to investigate the relevance of formulation of TCZ as polymeric mixed micelles (PMMs), containing Cremophor EL (acting as a penetration enhancer and a stabilizer), as a novel potential nano-system. The study aims to improve the dermal TCZ delivery via loading into PMMs enriched with Cremophor EL, which will consequently enhance the treatment of skin fungal infections upon topical application. Different variables affecting the PMMs’ characteristics were examined via application of a full factorial design (2^3^). Drug micellar incorporation efficiency, micellar size analysis, and zeta potential measurements of the prepared systems were conducted to study the effects of different variables. The PMMs belonging to the optimal formulation were visualized through transmission electron microscopy to reveal their inherent morphological properties and the physical stability of the nano-system was also evaluated. The permeation of TCZ from the optimal PMMs formulation was tested using an *ex vivo* study in comparison to the PMMs lacking Cremophor EL and drug suspension. In addition, *in vivo* skin deposition of TCZ from both the optimal PMMs and TCZ suspension was conducted in rats. Histopathological changes in rat skin receiving the optimal PMMs formulation were also assessed.

## 2. Materials and methods

### 2.1. Materials

Terconazole (TCZ) was kindly donated by Marcyrl Pharmaceutical Industries (Cairo, Egypt). Polyoxyethylene 20 sorbitan monooleate (Tween 80), acetonitrile (HPLC grade), methanol (HPLC grade), triethanolamine (HPLC grade), and glacial acetic acid (HPLC grade) were procured from Sigma-Aldrich Chemical Co. (St. Louis, MA). Pluronic F127, Pluronic P123, and Cremophor EL were purchased from BASF Co. (Florham Park, NJ). Disodium hydrogen phosphate, potassium dihydrogen phosphate, and sodium chloride were acquired from Merck (Darmstadt, Germany). Ethanol (95%) was obtained from El-Nasr pharmaceutical chemicals Co. (Cairo, Egypt). All other chemicals and solvents were of analytical grade and were used as received.

### 2.2. Preparation of TCZ loaded PMMs

A 2^3^ full factorial design was fashioned using Design-Expert^®^ software (Version 8, Stat-Ease Inc., Minneapolis, MN). The statistical procedure was carried out to analyze the experimental trials so that the optimal formulation could be successfully estimated. TCZ loaded PMMs were primed by varying the percentage of penetration enhancer (Cremophor EL) and using different weight ratios of both the drug to the Pluronic mixture and Pluronic P123 to Pluronic F127. PMMs were formulated according to the ethanol injection method reported earlier by Kakkar & Kaur (2011). In brief; specified amounts of TCZ and Pluronics mixture were weighed and dissolved in ethanol at 60 °C in an ultrasonic water bath sonicator (Crest Ultrasonics Corp.,NJ). The ethanolic mixture was then added to a predefined volume of aqueous solution of Cremophor EL, which was kept on a magnetic stirrer at 60 °C till the completion of ethanol volatilization. The formulation of TCZ loaded PMMs was accomplished through the formation of persistent clear solution.

With the aim of studying the influence of the penetration enhancer (Cremophor EL) on both the stability of the PMMs and their permeation characteristics through the skin; TCZ loaded PMMs were prepared by similar means; but lacking the Cremophor EL. The composition of the investigated formulae is shown in Table 1.

**Table 1.** Experimental runs, independent variables, and measured responses of the 2^3^ full factorial experimental design of PMMs.

PMM formulations	*X*_1_: weight ratio of total Pluronics to drug	*X*_2_: Weight ratio of Pluronic P123 to Pluronic F127	*X*_3_: percent of Cremophor EL in aqueous medium	*Y*_1_: MIE^a^ (%)	*Y*_2_: micellar size^a^ (nm)	*Y*_3_: PDI^a^	*Y*_4_: ZP^a^ (mV)
F 1	20	2	5	69.24 ± 8.12	27.99 ± 5.17	0.43 ± 0.42	− 7.65 ± 0.03
F 2	20	2	10	72.03 ± 6.66	72.35 ± 24.82	0.25 ± 0.04	− 5.69 ± 0.72
F 3	20	4	5	69.15 ± 1.33	90.55 ± 11.74	0.24 ± 0.09	− 5.11 ± 0.60
F 4	20	4	10	75.55 ± 10.39	162.42 ± 15.87	0.32 ± 0.05	−10.10 ± 1.70
F 5	40	2	5	51.87 ± 12.44	30.12 ± 4.88	0.57 ± 0.02	−10.46 ± 1.90
F 6	40	2	10	42.03 ± 6.89	97.29 ± 8.08	0.34 ± 0.07	− 6.35 ± 1.13
F 7	40	4	5	92.98 ± 0.4	33.23 ± 8.00	0.16 ± 0.00	− 9.65 ± 0.64
F 8	40	4	10	69.61 ± 7.35	25.40 ± 8.92	0.20 ± 0.05	− 4.59 ± 0.22
PMMs without Cremophor EL	40	4	0	92.98 ± 0.40	48.23 ± 15.07	0.16 ± 0.00	− 9.65 ± 0.64

a Data are represented as mean ± standard deviation (*n* = 3).

PPMs: polymeric mixed micelles; MIE: micellar incorporation efficiency percent; PDI: polydispersity index; ZP: zeta potential.

### 2.3. *In vitro* characterization of TCZ loaded PMMs

#### 2.3.1. Determination of TCZ micellar incorporationefficiency (MIE%)

Drug MIE% was calculated after quantifying the amount of incorporated drug, using the ultracentrifugation technique (Lopez-Pinto et al., 2005). To isolate the un-incorporated TCZ from the micelle-incorporated TCZ, a preset volume of the TCZ loaded PMMs samples were subjected to ultracentrifugation at 25,000 rpm at 4 °C for an hour using cooling ultracentrifuge (Sigma 3–30 KS, Sigma Laborzentrifugen GmbH, Germany). The concentration of TCZ in the supernatant was measured spectrophotometrically (Shimadzu, model UV-1601 PC, Kyoto, Japan) by measuring the UV absorbance at λ max 244 nm. Drug MIE% was calculated according to the following equation:

(1) $$MIE\%=\frac{Incorporated\;amount\;TCZ}{Total\;amount\;of\;TCZ}\times 100$$

#### 2.3.2. Determination of micellar size, polydispersity index (PDI), and zeta potential (ZP)

Mean micellar size and PDI of TCZ loaded PMMs were analyzed by photon correlation spectroscopy (PCS), using Zetasizer Nano ZS (Malvern Instruments, Malvern, UK), after appropriate dilution of the samples with distilled water. The ZP of the prepared systems was calculated on the basis of the electrophoretic mobility of formulated PMMs. The measurements were carried out by the same instrument used for particle size analysis. Before conducting the experiment, the samples were diluted with distilled water to bring the signal intensity through the limits mandatory by the instrument. All samples were analyzed in triplicates.

#### 2.3.3. Transmission electron microscopy (TEM)

The surface morphology of the optimal TCZ loaded PMMs was examined via TEM (Joel JEM 1230, Tokyo, Japan). Before sample visualization, the sample solution was loaded over the copper grid, and the surplus liquid was absorbed with a piece of filter paper, then the grid was dried in the air at room temperature. The copper grid was then negatively stained using saturated solution of uranyl acetate in 70% ethyl alcohol and dried in air at room temperature before being loaded in the microscope.

#### 2.3.4. Micelle stability

The storage stability of the optimal PMMs versus PMMs lacking Cremophor EL was experienced through the storage at room temperature for three months. The instability of the micelles was assessed by checking the changes in the physical characteristics of the nanomicellar solution; e.g. drug precipitation, turbidity of dispersion, and change in micellar size, in correlation with time (Gao et al., 2008).

### 2.4. *Ex vivo* studies

#### 2.4.1. Skin preparation

All the animal study protocols were approved by the Research Ethics Committee, Faculty of Pharmacy, Cairo University, Egypt. The dorsal skin was excised from newly born rats weighing 70 ± 20 g after being sacrificed. The dermal surface was carefully cleaned to remove subcutaneous tissues and adhering fats without damaging the epidermal surface.

#### 2.4.2. *Ex vivo* permeation study

Permeation of TCZ, through excised rat skin, from the optimal PMMs, PMMs lacking Cremophor EL, and TCZ aqueous suspension was assessed. The skin was mounted on a diffusion cell such that the stratum corneum was facing the donor compartment. The membrane surface area available for diffusion was 0.636 cm^2^. The donor side was charged with 0.1 mL of one of the tested formulations (10 mg/mL) while the receptor compartment was filled with 20 mL of phosphate buffer saline (PBS of pH 7.4) containing 0.5% Tween 80 at 32 ± 0.5 °C and stirred at 50 rpm. At various time intervals up to 8 h, samples from the receptor fluid (0.5 mL) were withdrawn and replaced immediately by fresh buffer solution to maintain both constant volume and sink conditions. The withdrawn samples were then analyzed using a validated HPLC method. For each formulation, the amount of drug permeated per unit area (µg/cm^2^) was plotted versus time (h). The flux at 8 h (*J*_max_) was computed using the following equations (El Zaafarany et al., 2010):

(2) $$J_{max}=\frac{Amount\;of\;drug\;permeated\;}{Time*Area\;of\;the\;membrane}$$

At the end of the permeation study, the skin was taken from the diffusion cell and washed with 10 mL of normal saline to remove the adhering drug. The skin was then cut into small pieces and sonicated in 5 mL methanol for 30 min using bath sonicator to leach out the deposited drug. The samples were centrifuged and the supernatant was passed through 0.45 mm membrane filter and assayed by HPLC. In addition, the local accumulation efficiency (LAE) values for TCZ were calculated as the ratio of the amount drug accumulated into the skin to that permeated through the skin at the end of the experiment. The differences in the values of *J*_max_, amount of deposited TCZ and LAE were statistically assessed by one-way ANOVA using SPSS 19.0 software^®^ (SPSS Inc., Chicago, IL). *Post-hoc* analysis was done using Tukey’s HSD (honest significant difference) test. The difference at *p* ≤ .05 was considered significant.

#### 2.4.3. HPLC determination of TCZ

An isocratic, previously validated HPLC method was used for the quantification of TCZ (Abdelbary et al., 2016). The HPLC system consisted of a Zorbax Extend- C18 column (4.6 mm × 250 mm) containing 3.5 mm size adsorbent as stationary phase (Agilent technologies, Santa Clara, CA), LC-10AD pump, SPD-10 A UV detector, and CR6A Chromatopac integrator (Shimadzu, Kyoto, Japan). The column was maintained at room temperature (25 ± 2.0 °C). The mobile phase consisted of a mixture of acetonitrile, an aqueous solution of disodium hydrogen phosphate (20 mM), and triethanolamine (60:39.8:0.2 v/v/v, respectively) and the pH was adjusted to 4.0 with glacial acetic acid. Elution was carried out at a flow rate of 1 mL/min. Effluents were monitored at 254 nm. Under the described conditions, TCZ was eluted at 4 min.

### 2.5. *In vivo* studies

#### 2.5.1. Experimental animals

A total of 48 male Wistar rats, weighing 150–200 g, were involved in the *in vivo* studies. The animals were supplied with standard diet and tap water *ad libitum* and placed individually in cages with wide mesh wire bottoms to avoid coprophagy. Thirty six animals were included in the *in vivo* skin deposition study while twelve animals were involved in the histopathological study.

#### 2.5.2. *In vivo* skin deposition studies

Before conducting the experiment, the rats were randomly separated into two groups with 18 animals in each group. Bottle caps that served as drug pools with an area of 4.91 cm^2^ were fixed to rat dorsal skin which was shaved to remove hair with an electric clipper 24 h before application of the sample. Half milliliter of the TCZ tested formulation (the optimal TCZ loaded PMMs or TCZ aqueous suspension) was added non-occlusively into the drug pool. The groups were treated according to the following pattern: group I: topical application of TCZ loaded PMMs and group II: topical application of TCZ suspension. After different time intervals of application of the treatments (1, 2, 4, 6, 8, and 24 h), three animals from each group were humanely sacrificed using an overdose of anesthetic ether (Shen et al., 2014) and the dorsal rat skin that was in contact with the formulation was excised then immediately washed with 10 mL of normal saline in two divided portions. The excised skin sections were cut into pieces and sonicated in 5 mL methanol for 30 min. The skin homogenate was then filtered through a 0.45 mm membrane filter and the concentration of TCZ was determined using HPLC method as mentioned earlier. The obtained data were used to calculate the skin deposition of TCZ generated by the tested formulations. Statistical analysis was performed using t-test and the difference was considered to be statistically significant at *p* ≤ .05.

#### 2.5.3. *In vivo* histopathological study

*In vivo* histopathological study was conducted to assess the irritation potential and observe the ultrastructural alterations in the skin upon exposure to TCZ loaded PMMs. The rats were randomly divided into two groups with six animals in each group. Group I acted as control while animals in group II were treated topically with TCZ loaded PMMs, onto the skin surface three times daily for a period of one week. The animals were then sacrificed and the skin was excised for histopathological investigation according to the procedures reported by Bancroft et al. (2007). Briefly, skin samples from different rats groups were fixed in 10% formal saline for 24 h then washed with water and dehydrated by serial dilutions of alcohols (methyl alcohol, ethyl alcohol, and absolute ethyl alcohol). Then, specimens were cleared in xylene and embedded in paraffin bees wax blocks and kept at 56 °C for 24 h. Sections from the paraffin blocks of 4 mm thickness were cut using a microtome (Leica Microsystems SM2400, Cambridge, England), deparaffinized, stained with hematoxylin and eosin and then observed under a light microscope.

## 3. Results and discussion

### 3.1. Preparation of PMMs

A mono-micellar system is known to have many drawbacks namely; low drug loading, larger particle size and low stability, that can be ameliorated by mixing different polymeric surfactants to produce mixed micellar systems (Attia et al., 2011). For this purpose, the prepared systems, herein, were prepared by using two types of Pluronics of different hydrophilic-lipophilic balance (HLB) that is reported also to aid in reaching the optimum thermodynamic and kinetic stabilities for the formed micelles (Dutra et al., 2015). Low HLB Pluronic (Pluronic P123 with HLB = 7–9) is known to increase the thermodynamic stability of the micelles due to the tight hydrophobic interactions of its propylene oxide blocks while the high HLB Pluronic (Pluronic F127 with HLB = 22) possesses long hydrophilic chain that increase the kinetic stability due to steric hindrance for micelle aggregation (Lee et al., 2011). Herein, Cremophor EL (HLB = 12–14) was incorporated in PMMs to evaluate its effect on the stability of the mixed micelles nanosystems as well as its ability to enhance the introduction of the drug into skin layers.

### 3.2. Analysis of factorial design

The selected factors in the current study and their levels were based on preliminary trials (data not shown) to specify the expected ranges of the independent variables. The design applied here was a full factorial design (2^3^) with statistical analysis through Design-Expert^®^ software. The predicted *R*^2^ was interpreted as a measure of the precision of a predictable model response value (Chauhan & Gupta, 2004, Kaushik et al., 2006). The adjusted and predicted *R*^2^ values should varied in the range of 0.20 from each other to be rationally matched (Annadurai et al., 2008). In our study, the predicted *R*^2^ values were in line with the adjusted *R*^2^ in all responses except PDI and ZP (data are not shown).

#### 3.2.1. Effect of formulation variables on MIE%

**Figure 1.**
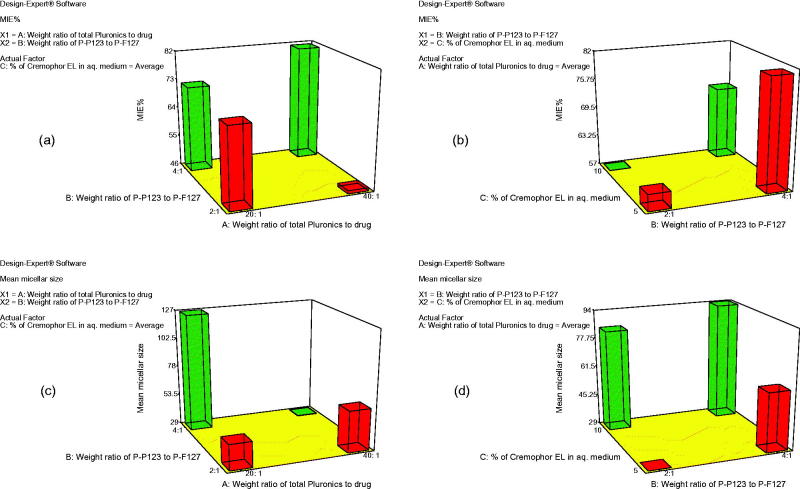
3-D plot model graphs demonstrating the effect of factors; *X*_1_: weight ratio of total Pluronics to drug, *X*_2_: weight ratio of Pluronic P123 to Pluronic F127, and X_3_: percent of Cremophor EL in aqueous medium on micellar incorporation efficiency percent (MIE%) (a and b) and mean micellar size (c and d).

Pluronics known by an elevated hydrophobicity like Pluronic P123, are ideal for the enhancement of the solubilization of poorly soluble drugs. But unfortunately, when used alone in drug delivery, it was reported to generate large lamellar structures, in addition to, a stability problem that lead to aggregates formation in aqueous media (Oh et al., 2004). So different weight ratios of two types of Pluronics (P123 and F127) were used and Cremophor EL was also added to check its additional effect in producing more promising TCZ nanomicellar delivery system, PMMs. For the sake of preparing a successful nano-carrier for TCZ, the mixed micelles have to entrap significant amount of the drug. The percent of MIE of the prepared PMMs ranged from 42.03 to 97.03%, as shown in Table 1. The influence of the weight ratio of total Pluronics to TCZ (*X*_1_), weight ratio of Pluronic P123 to Pluronic F127 (*X*_2_), and the percentage of Cremophor EL (*X*_3_) on the MIE% of PMMs is graphically illustrated as 3-D plots, Figure 1(a,b).

Factorial analysis of variance showed that both *X*_1_ and *X*_3_ had insignificant effect on MIE% (*p* = .0920 and *p* = .1590, respectively). On the other hand, the weight ratio of Pluronic P123 to Pluronic F127 (*X*_2_) significantly affected the MIE% (*p* = .0013). Statistical analysis showed that upon increasing the ratio of Pluronic P123 to Pluronic F127 from 2:1 to 4:1, the MIE% had profoundly increased. The hydrophilic block, polyethylene oxide (PEO) units, of Pluronic F127 is approximately five times larger than that of Pluronic P123 which can explain the higher MIE% obtained upon increasing the ratio of the more lipophilic Pluronic P123 that provided more hydrophobic pool to entrap the water-insoluble drug, TCZ (Attwood et al., 2007). These results are matched with the findings of Kulthe et al. (2011) where, the MIE% of two different formulations were compared and the mixed micelle containing the higher number of the hydrophobic propylene oxide (PPO) units showed 94.05 as MIE% of the insoluble drug relative to those containing lesser hydrophobic units (57.60%).

#### 3.2.2. Effect of formulation variables on the mean micellar size

Particle size is considered as one of the most important factors controlling the skin penetration of the drug delivery system (Verma et al., 2003). The development of a nano-sized system ensures deeper penetration into skin strata (du Plessis et al., 1994). The effect of different formulation variables, namely the weight ratio of total Pluronics to drug (*X*_1_), the weight ratio of the two types of Pluronics (P123:F127) (*X*_2_), and the percentage of Cremophor EL (*X*_3_) on the mean micellar size of the prepared TCZ PMMs, was assessed and graphically illustrated in Figure 1(c,d). Z-average diameter which is representative for the mean hydrodynamic diameter of the micelles was used to describe the mean micellar size (Das et al., 2012). The hydrodynamic diameters of the prepared PMMs were in the nanometric range of 25.40 ± 8.92 to 162.42 ± 15.87 nm.

The analysis of variance (ANOVA) data obtained from the formed PMMs showed that weight ratio of the total Pluronics to drug (*X*_1_) had significant effect on the mean micellar size (*p* = .003). As the weight ratio of total Pluronics to drug increased from 20:1 to 40:1, the particle size significantly decreased, and this could be attributed to the solubilization phenomena exhibited by the micellar dispersion (Shinoda et al., 2016). Although, hydrophobic Pluronics were known to produce larger aggregates compared to hydrophilic ones that usually generate smaller micelles in aqueous environment (Das et al., 2012), the change in the weight ratio of Pluronic P123 to Pluronic F127 from 2:1 to 4:1 (*X*_2_) in the current research didn’t significantly affect the micellar size (*p* = .0746).

On the other hand, the percentage of Cremophor EL (*X*_3_) showed significant effect on the micellar size (*p* = .0022), where an increment in Cremophor EL content had increased the micelles size. Cremophor EL is categorized as a nonionic surfactant assembled of a hydrophobic tail (glycerol polyethylene glycol ricinoleate and other fatty acid esters of polyethylene glycol), and a hydrophilic head (free polyethylene glycols and ethoxylated glycerol). Herein, Cremophor EL acts as a stabilizer and this role is related to the balance between the hydrophobic moiety that is become incorporated in the tail region and the hydrophilic domain that is oriented towards the aqueous medium. Consequently, increasing the percentage of Cremophor EL led to larger mean micellar size through formation of larger shield which will provide steric stabilization of the PMMs and prevent their aggregation (Chong et al., 2012; Madheswaran et al., 2014).

#### 3.2.3. Effect of formulation variables on ZP and PDI of the prepared PMMs

The ZP values for PMMs are illustrated in Table 1. All prepared formulations had ZP values within the range of −4.59 ± 1.22 to −10.46 ± 1.90 mV. The relatively low observed ZP values of the prepared dispersions could be attributed to the outward shift of the slipping plane, at which ZP is measured, which was caused by thick adsorbed polymer layer on the surface (Abdelbary et al., 2015). It is well known for triblock copolymers (Pluronics) that the hydrophobic PPO block anchors to the hydrophobic surface, leaving the PEO chains extending in the aqueous phase, leading to the formation of a brush conformation. According to Gouy-Chapman theory, the slipping plane is moved to a point further out from the surface where the charge density is much smaller than on the surface resulting in lower ZP.

PDI value of 0 describes a completely mono-dispersed particles population, in contrast, a value of unity indicates greatly poly-dispersed particles (Zeisig et al., 1996). The formed PMMs formulations had PDI ranged from 0.157 to 0.425 which indicates narrow size distribution and excellent homogeneity, Table 1. Factorial analysis of variance indicated insignificant effect (*p* < .05) for the three variables, namely; the weight ratio of total Pluronics to drug (*X*_1_), the weight ratio of the two types of Pluronics (P123:F127; *X*_2_) and the amount of Cremophor EL (*X*_3_), on both ZP and PDI measurements.

#### 3.2.4. Selection of the optimal formulation

The eight formulations data were analyzed using Design-Expert^®^ software following a full factorial design aiming at the selection of the optimal PMMs formulation. The set of factors contributing to the choice of the optimum formulation were; attaining maximum values of MIE%, and minimum values for micellar size. It should be stated that PDI and ZP were not taken into consideration, as they were not affected by the three formulation variables (Nour et al., 2016). So based on the previously discussed results, F7 (PMMs formulation in which the ratio of total Pluronics to drug was 40:1, the weight ratio of Pluronic P123 to Pluronic F127 was 4:1 and percentage of Cremophor EL in aqueous phase was 5%) was chosen as the optimal PMMs formulation. This formulation showed MIE % of 92.98 ± 0.40%, mean micellar size equals of 33.23 ± 8.00 nm, very small PDI value of 0.16 ± 0.00, and ZP of −9.65 ± 0.64 mV. Therefore, it was chosen as the most successful PMMs for further investigation in the current research.

### 3.3. Transmission electron microscopy (TEM)

**Figure 2.**
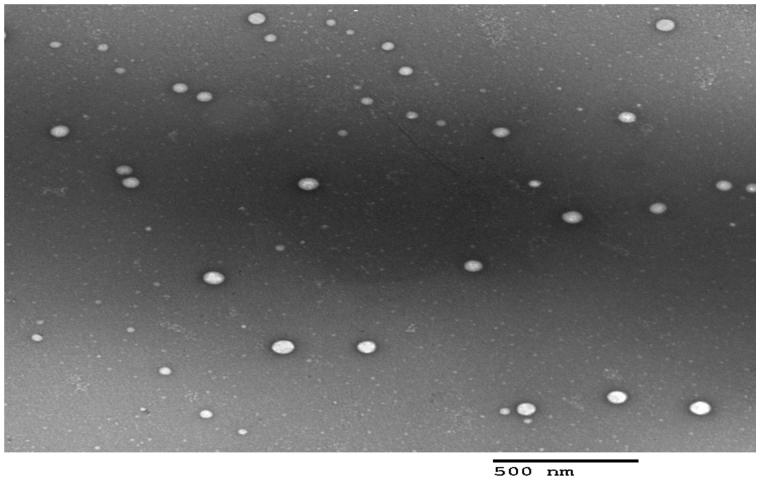
Transmission electron micrograph of the optimal PMMs formulation (F7).

TEM examination helps to confirm the obtained results from Malvern particle size analyzer, in addition to, describing the morphology of the prepared system (Al-mahallawi et al., 2015). The TEM examination results revealed that all micelles were spherical in shape with good dispersibility. There was no aggregation as shown in Figure 2. As appeared in the TEM micrograph, the mean size of the micelles was in good agreement with the size obtained from Malvern particle size analyzer.

### 3.4. Micelle stability

The optimal TCZ loaded PMMs, F7, and the PMMs of the same composition but lacking Cremophor EL were subjected to stability study. The results showed that F7 was stable during storage at room temperature (25 °C ) for three months. No precipitation of drug and no significant change in the micelle size and size distribution were observed during this period. On the other hand, PMMs formulation prepared without Cremophor EL was only stable for one month, where precipitation of the drug and turbidity of the dispersion were noticed beyond this period of time. As previously mentioned, incorporation of Cremophor EL led to the enhancement in the steric stability due to the steric shield formed by its hydrophilic domains (Chong et al., 2012, Madheswaran et al., 2014).

### 3.5*. Ex vivo* skin permeation studies

**Figure 3.**
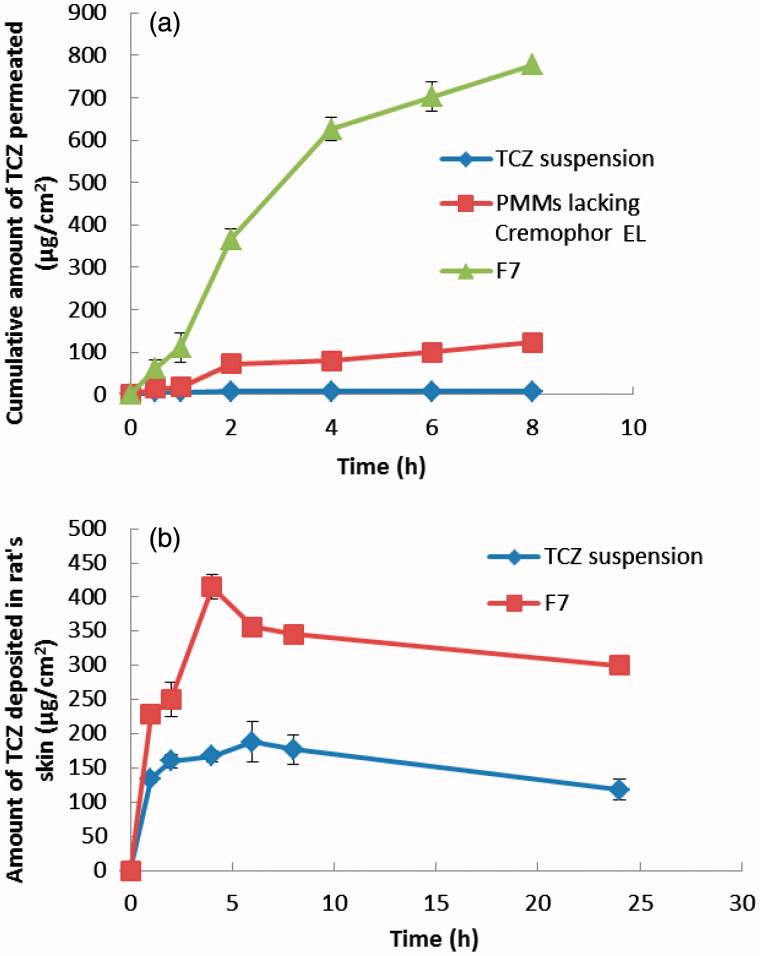
(a) Cumulative amount of TCZ permeated per unit area across skin via optimal PMMs formulation (F7) compared to PMMs lacking Cremophor EL and TCZ suspension. (b) Cumulative amount of TCZ deposited per unit area in the skin via optimal PMMs formulation (F7) compared to TCZ suspension.

It is challenging to evolve a simple formulation that enables drug release into the skin. So the *ex vivo* skin permeation test was performed since it is known to give great idea about the *in vivo* behavior of a newly developed dermal drug delivery system. The cumulative amount of TCZ permeated per unit area across excised rat skin as a function of time obtained from the optimal PMMs formulation (F7) and PMMs lacking Cremophor EL relative to drug suspension is illustrated in Figure 3(a). The values of *J*_max_, the amount of drug deposited in the skin and LAE are listed in Table 2. It was clear that very poor permeation capability has been obtained from the TCZ suspension, while the PMMs formulation with no Cremophor EL in its composition had shown an intermediate permeation profile. TCZ is permeated the most via the optimal PMMs formulation, F7. Based on the observed permeation profiles as well as the different calculated parameters, it can be concluded that the loading of TCZ into PMMs (either containing Cremophor EL or not) resulted in a significant improvement (*p* < .05) in the dermal drug delivery relative to the drug suspension, with LAE values of 3.90 ± 0.24, 2.15 ± 0.17 and 0.44 ± 0.10 for F7, PMMs lacking Cremophor EL, and TCZ suspension, respectively. These results could be attributed to the penetration enhancing capabilities of the included surfactants in the construction of PMMs, which are known by their ability to solubilize the stratum corneum lipids. Keratin interactions are considered as additional mechanism through which the surfactants can exert penetration-enhancing effects (Ayala-Bravo et al., 2003). However, the obtained results also clarify the superiority of F7 over the PMMs lacking Cremophor EL. This further improvement in skin delivery of F7 relative to the PMMs lacking Cremophor EL can be explained by the role of Cremophor EL incorporated in the F7 formulation. This additional surfactant will augment the interaction of the prepared PMMs with keratin in the corneocytes. It is hypothesized that surfactants generally have the ability to enter into the intracellular matrices of the stratum corneum and to interact and bind to keratin filaments, which will lead to an elevation in the penetration ability and therefore an enhancement in the skin permeability (Date & Patravale, 2007). Furthermore, Cremophor EL has the ability to increase the epithelial permeability by disturbing the cell membrane. It can also reversibly open the tight junctions in the epithelial wall facilitating the drug transport across the membrane (El-Zahaby et al., 2017). So the Cremophor EL based PMMs, F7 had successfully enhanced TCZ cutaneous permeation in comparison to PMMs lacking Cremophor EL, and TCZ suspension, thus ensuring a promising topical delivery of the drug.

**Table 2.** Parameters obtained from *ex vivo* studies for the optimal PMMs (F7), PMMs lacking Cremophor EL, and TCZ suspension.

Formulation	Flux at 8 h (*J*_max_) (µg/cm^2^/h)^a^	Amount of drug deposited in the skin after 8 h (µg/cm^2^)^a^	Local accumulation efficiency (LAE)^a^
TCZ suspension	0.92 ± 0.05	3.20 ± 0.13	0.44 ± 0.10
Optimal PMMs (F7)	32.40 ± 1.46	1012.16 ± 38.46	3.9 ± 0.24
PMMs without Cremophor EL	15.32 ± 0.93	264.25 ± 12.52	2.15 ± 0.17

a Each value represents mean ± standard deviation (*n* = 3).

### 3.6. *In vivo* studies

#### 3.6.1*. In vivo* skin deposition

The skin deposition is a measure of the ability of the drug delivery system to penetrate the skin layers and reach its site of action. The results of the *in vivo* skin deposition of TCZ from the optimal PMMs formulation (F7) in comparison with TCZ suspension are presented in Figure 3(b). It is clear that TCZ loaded PMMs had presented higher amounts of TCZ deposited in the skin compared to the TCZ suspension. The calculated [AUC]_0_^24^ for F7 was significantly higher (*p* < .05) than that of the drug suspension (7687.855 ± 155.171 and 3622.133 ± 170.239 µg h/cm^2^, respectively). In other words, the selected PMMs achieved 2.12 folds increase in the AUC compared to the drug suspension. These results demonstrate the ability of prepared PMMs to carry the drug molecules into the skin. Hence, it could be concluded that PMMs effectively improved the skin deposition of TCZ, demonstrating the potential of these nano-carriers to overcome the stratum corneum barrier, and concentrate the drug within the skin layers following topical application.

#### 3.6.2. Histopathological examination

**Figure 4.**
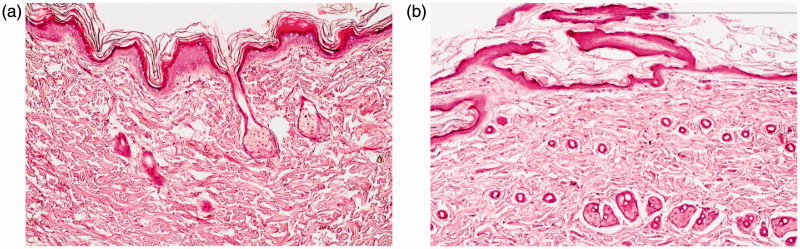
Photomicrographs showing histopathological sections (hematoxylin and eosin stained) of (a) normal untreated rat skin and (b) rat skin treated with optimal PMMs formulation (F7).

The stained rat skin was examined under light microscope where; the untreated control group (Group I) showed normal skin structure, Figure 4(a).  There was no significant alteration in the integrity of both the epidermis and dermis. Moreover, no inflammatory cells were observed. Regarding Group II, treated daily with the application of the optimal TCZ loaded PMMs (F7), the micrograph shows neither inflammation nor skin irritation, Figure 4(b). The skin structure was normal and the underlying dermal connective tissue was intact. However, small changes were observed in the keratinized layer and these changes could be attributed to the composition of PMMs (surfactants effect). As a conclusion, TCZ loaded PMMs were safe to be used topically with no major irritation or inflammation consequences.

## 4. Conclusion

Skin delivery of anti-fungal drugs is preferred in many cases owing to its fineness over oral treatment in terms of avoiding toxic side effects and the risk of drug interactions, in addition to its privilege in targeting the site of infection. In this study, PMMs enriched with Cremophor EL were successfully fabricated by ethanol injection method as novel, physically stable nano-carriers for the topical delivery of TCZ. A complete 2^3^ factorial design was utilized for the formulation of the PMMs. The optimal PMMs formulation showed small mean micellar size, spherical morphology, very high drug MIE%, and better physical stability when compared with PMMs lacking Cremophor EL. The performed *ex vivo* study revealed the superiority of the investigated PMMs compared to PMMs lacking Cremophor EL and aqueous drug suspension. The *in vivo* studies demonstrated that the optimal PMMs exhibited a greater extent of TCZ deposition in the rat dorsal skin relative to TCZ suspension. Furthermore, the *in vivo* histopathological studies confirmed the non-irritant nature of the applied PMMs. Accordingly, the use of PMMs enriched with Cremophor EL may be considered as a promising approach for enhancing topical delivery of TCZ and thus, providing successful local treatment of skin fungal infections.
